# Neuromodulators in Acute and Chronic Cough in Children: An Update from the Literature

**DOI:** 10.3390/ijms252011229

**Published:** 2024-10-18

**Authors:** Simone Foti Randazzese, Fabio Toscano, Antonella Gambadauro, Mariarosaria La Rocca, Giulia Altavilla, Mariagrazia Carlino, Lucia Caminiti, Paolo Ruggeri, Sara Manti

**Affiliations:** 1 Pediatric Unit, Department of Human Pathology in Adult and Developmental Age “Gaetano Barresi”, University of Messina, Via Consolare Valeria, 1, 98124 Messina, Italy; simone.fotirandazzese@studenti.unime.it (S.F.R.); fabio.toscano1@studenti.unime.it (F.T.); mariarosaria.larocca@studenti.unime.it (M.L.R.); giulia.altavilla@studenti.unime.it (G.A.); lcaminiti@unime.it (L.C.); 2 Pediatric Unit, Department of Medical and Surgical Sciences, Magna Graecia University of Catanzaro, 88100 Catanzaro, Italy; mariagrazia.carlino@studenti.unicz.it; 3 Pulmonology Unit, Department of Biomedical, Dental, Morphological and Functional Imaging Sciences (BIOMORF), University of Messina, Via Consolare Valeria, 1, 98124 Messina, Italy; paolo.ruggeri@unime.it

**Keywords:** acute cough, chronic cough, neuromodulators, review

## Abstract

Cough is one of the most common reasons leading to pediatric consultations, negatively impacting the quality of life of patients and caregivers. It is defined as a sudden and forceful expulsion of air from the lungs through the mouth, typically triggered by irritation or the stimulation of sensory nerves in the respiratory tract. This reflex is controlled by a neural pathway that includes sensory receptors, afferent nerves, the brainstem’s cough center, efferent nerves, and the muscles involved in coughing. Based on its duration, cough in children may be classified as acute, lasting less than four weeks, and chronic, persisting for more than four weeks. Neuromodulators have shown promise in reducing the frequency and severity of cough by modulating the neural pathways involved in the cough reflex, although they require careful monitoring and patient selection to optimize the outcomes. This review aims to examine the rationale for using neuromodulators in the management of cough in children.

## 1. Introduction

Cough is defined as a forced expulsive maneuver, usually against a closed glottis, accompanied by a distinctive sound [1]. Coughing is a protective physiological reflex, helping to remove secretions and/or foreign bodies from the airways [2]. However, cough may also be considered a non-specific symptom and signal the presence of conditions affecting the upper or lower respiratory tract, requiring prompt diagnosis and treatment [3]. Based on its quality, cough may be wet/productive, involving mucus production, or dry, which is often irritative [4,5].

In pediatric age, according to the duration, it may be categorized as acute, which lasts less than 4 weeks, and chronic cough, which persists for more than 4 weeks [6,7]. Most children experience episodes of acute cough, often due to viral upper respiratory tract infections (URTIs) [8]. On average, a preschool-aged child may experience 6 to 8 viral respiratory infections per year, each accompanied by coughing [9]. Chronic cough affects approximately 5% to 10% of children [10]. The prevalence varies based on geographic location, environmental factors (e.g., exposure to tobacco smoke or allergens), and underlying health conditions [10]. It commonly results from post-infectious cough, asthma, protracted bacterial bronchitis (PBB), and conditions like gastroesophageal reflux disease (GERD) [11].

There are little population-based data on the epidemiology of cough in children and how this varies by age and sex, or between children with and without wheeze. Jurca et al. conducted a study using a population cohort from Leicestershire, UK, to investigate the prevalence of cough from early childhood through adolescence by administering repeated questionnaires. They found that 10% of children coughed more than their peers, 69% coughed with colds, 34–55% coughed without colds, and 25% experienced night-time cough. Cough prevalence varied by age, with boys coughing more in early childhood, but this trend reversed after age 14. Cough was more frequent in children with wheeze. The study highlights that cough prevalence differs based on age, sex, and the specific questions asked [12]. More recently, a systematic review by Bergmann et al. examined studies on the prevalence, causes, and prognosis of cough in children consulting primary care physicians. The key findings include the following: (1) Cough is a common reason for medical visits, with prevalence ranging from 4.7% to 23.3% of all consultations, and up to 60% when considering specific encounters. Cough is more frequent in children than adults, especially in younger age groups and less common in summer. (2) Acute cough is typically caused by URTIs (62.4%) and bronchitis (33.3%), while chronic cough is often due to recurrent respiratory infections (RRIs) (27.7%), asthma (up to 50.4% for cough lasting over 3 weeks), and pertussis (37.2%). Serious conditions like croup, pneumonia, and tuberculosis are rare. (3) In chronic cases, cough may last from 24 to 192 days, with 62.3% of children still coughing two months after symptoms begin [13].

Cough may significantly impact a child’s quality of life (QoL), leading to sleep disorders, school absences, and stress for both the child and his caregivers [14]. Traditional treatments focus on addressing the cause of the cough; however, cough sometimes remains refractory despite appropriate therapy [6,7].

Neuromodulators, which influence the neural pathways involved in cough reflex regulation, are emerging as a potential therapeutic option for managing both acute and chronic cough, especially when conventional treatments fail [15].

This review aims to explore the role of neuromodulators in pediatric cough management, examining their efficacy, safety, and place within the broader context of treatment strategies for acute and chronic cough in children. An accurate review of the current literature was conducted using the PubMed database, focusing on a critical assessment without the application of statistical analyses or standardized methodologies. Key terms such as “cough” OR “acute cough” AND “chronic cough” AND “neuromodulators” AND “children” OR “pediatric age” OR “pediatrics” were included in the search. Medical Subject Headings (MeSH) terms were used alongside free-text searches to ensure comprehensive coverage of the relevant literature. The analysis included reviews, systematic reviews, meta-analyses, observational studies, randomized controlled trials (RCTs), and evidence-based guidelines of the major scientific societies up to 31 July 2024.

## 2. Cough: A Vital Reflex

Cough is a vital reflex protecting the respiratory tract from inhaling foreign bodies and excessive secretions [2]. As a physiological reflex, healthy children usually cough on average 11 times daily (range 1–34) [16].

**Figure 1:**
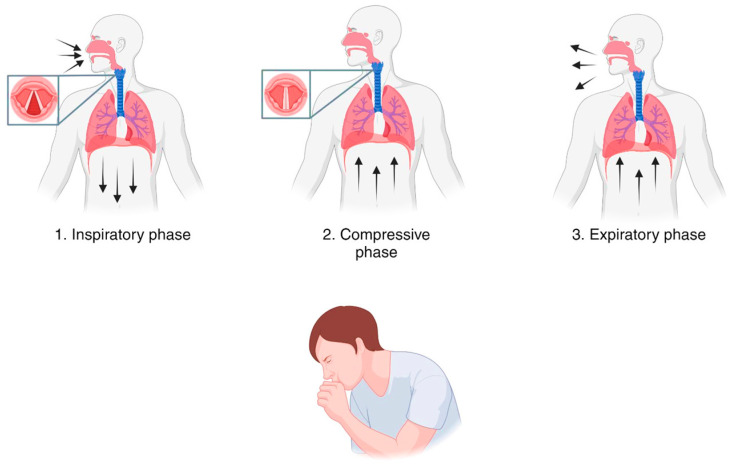
The three different phases of the cough reflex.

Cough can occur voluntarily or in response to endogenous (e.g., mucus, refluxate, or inflammation) or exogenous (e.g., cold air, particulates, or smoke) stimuli [17]. Irritants stimulate receptors and ion channels (e.g., Transient Receptor Potential Vanilloid 1 (TRPV1), Transient Receptor Potential Ankyrin 1 (TRPA1), TRPV4, and Transient Receptor Potential Melastatin 8 (TRPM8)) localized on vagal afferent nerve terminations throughout the larynx, conducting airways, alveolar septa, and lung parenchyma [18,19]. Cough receptors are also present in other areas, including the pharynx, external auditory canals, eardrums, paranasal sinuses, pleura, diaphragm, pericardium, and esophagus [19,20]. The afferent impulses go to the medulla oblongata, where they are processed in the cough center, located in the upper brain stem and pons. Then, efferent impulses travel via the vagal, phrenic, and spinal motor nerves to the diaphragm, abdominal wall, and muscles. The inspiratory and expiratory muscles receive impulses from the nucleus retroambigualis by phrenic and other spinal motor nerves. The larynx receives impulses from the nucleus ambiguous by the laryngeal branches of the vagus [20]. The result of this reflex pathway is the cough reflex arc, which mediates three different phases of cough (Figure 1):

1. Inspiratory phase, which consists of a rapid inhalation after the opening of the glottis by contraction of the arytenoid cartilage. This phase involves 50% of vital capacity with wide variations due to the nature of the stimulus and the type of involved receptor.

2. Compressive phase, with a prompt closure of the glottis and adduction of the vocal cords following the contraction of the adductor muscles of the arytenoid cartilages. At the same time, the intrapulmonary pressure increases due to the vigorous contraction of the abdominal muscles and other expiratory muscles which induce the compression of the alveoli and bronchioles.

3. Expiratory phase, which results in an explosive expiration of air from the lungs to the outside due to the sudden opening of the vocal cords and epiglottis for action of the abductor muscle of the arytenoid cartilages. Then, the expiration continues in a passive manner, favored by the complete relaxation of the diaphragm [2,21].

The cell bodies of the vagal sensory nerves are mostly located in the nodose and jugular ganglia, although about 1% originate from the thoracic dorsal ganglia [22]. There are several sensory nerve subtypes in the airways which can be divided into mechanosensitive and chemosensitive [23]. The mechanosensitive subtypes are important for clearing the airways of inhaled or aspirated foreign bodies and are represented by the rapidly adapting receptors (RARs) and the slowly adapting receptors (SARs) [24]. The chemo-sensitive subtypes, which evoke cough in response to inhaled noxious chemicals or endogenously-released mediators derived from pathological processes, are represented by the Aδ fibers and the C fibers [25,26,27]. RARs are myelinated fibers activated by stimuli which evoke bronchospasm and mucus production, and by substances such as histamine, capsaicin, substance P, and bradykinin [24,28,29]. RARs can cause cough even under anesthesia, allowing the body to defend the lungs from harmful particles [30,31]. SARs are primarily distributed in the alveoli and bronchioles, and occasionally associated with the bronchiolar smooth muscles. They are highly sensitive to the mechanical forces put on the lungs during breathing, especially during inspiration and prior to the initiation of expiration, and they are important in the Hering–Breuer reflex [20,32]. Aδ fibers are fast-conducting myelinated fibers originating in the jugular ganglia and are activated by TRPV1 agonists, such as capsaicin [33,34]. C fibers are mostly nerve fibers innervating the airways and are found predominantly in the airway epithelium [35]. They are unmyelinated and slow conducting fibers; these fibers are responsible for the conscious perception of irritants and are blocked by anesthesia [22]. C fibers are activated by agonists of TRPV1 and TRPA1 (e.g., acrolein and cinnamaldehyde) [26,30]. 

Receptors and ion channels have a pivotal role in the initiation and regulation of cough [36]. TRPV1 and TRPA1 are members of the transient receptor potential (TRP) superfamily which are cation-selective ion channels with a preference for the calcium ion (Ca^2+^) [37]. TRP channels act as cellular sensors and can be stimulated by different triggers, such as arachidonic acid derivatives, a low airway pH or high proton concentration, reactive oxygen species (ROS), oxidative stress end-products, altered osmolarity, and changes in temperature and stretch [38,39]. TRPV1 was the first TRP channel to be described as a key regulator of tussive reflexes [40] and it is activated by multiple stimuli, such as irritant chemicals (e.g., capsaicin), low pH, high temperature (≥43 °C), and inflammatory mediators (bradykinin, prostaglandin E2 (PGE2), and protease-activated receptors (PARs)) [38,41,42,43,44,45]. TRPV1 is mainly located in C fibers and in a small subset of thin myelinated Aδ fibers. TRPV1-positive neural fibers innervate the respiratory tract, from the upper to the lower airways, but its density is highest in the proximal airways and progressively decreases in more distal tracts [46,47]. TRPA1 was initially described as a noxious cold sensor and mechanosensory, but it is activated by several stimuli, such as the end-products of oxidative stress (e.g., hypochlorite, nitric oxide, and hydrogen peroxide), PGE2, bradykinin, and PAR2 [30,48,49,50,51]. It is expressed in airway vagal neurons and in non-neuronal cell types, including epithelial cells, airway smooth muscle cells and fibroblasts [52,53]. TRPA1 seems to cause cough by the activation of the vagal bronchopulmonary C fibers in the lungs [54,55]. TRPV4 and TRPM8 are also members of the TRPV superfamily, and their role in cough modulation was recently reported. TRPV4 was firstly described as a sensor of osmotic stress, but it is activated by moderate temperatures (>24 °C), PAR2, and hypotonic solutions [56,57]. It is mainly located in the central and peripheral nervous systems and causes cough through the activation of mechanosensitive Aδ fibers [58,59]. TRPM8 is a thermosensor activated by cool temperatures (between 15 and 28 °C) and other compounds, such as menthol, icilin, and eucalyptol [41,60]. It is expressed in a subpopulation of cold-responsive primary afferent neurons within the dorsal root and trigeminal ganglia, and in neuronal afferents located in the upper and lower airways [41,49,61,62]. TRPM8 is responsible for cough and bronchoconstriction induced by the inhalation of cold air, but menthol—one of its agonists—seems to have antitussive properties [61,63,64].

Distinct from reflex cough, voluntary cough is consciously achieved by the prefrontal cortical control of descending motor pathways, without any cough-related peripheral sensory nerve activity from the airways [65]. It is supposed that cough associated with URTIs is mainly elicited by the voluntary pathway as a cognitive recognition of the necessity to protect the airways by the so-called urge to cough (UTC). URTIs, by pharyngeal inflammation, stimulates the cerebral cortex and elicits cough voluntarily [66]. This mechanism explains why cough associated with URTIs may be poorly responsive to codeine and common antitussive synthetic drugs, which usually interact with the reflex cough pathway [67]. Moreover, cough may be voluntarily modulated or inhibited by acting on the cerebral cortex [68]. It has been demonstrated that a placebo treatment can reduce the cough frequency in patients with URTIs by 50% by interacting with the voluntary component of cough through the generation of endogenous opioids [69]. The comprehension of these mechanisms is essential to understand the pathophysiology of acute and chronic conditions related to cough and to recognize the basis of their potential neuromodulation.

## 3. Acute Cough

Cough is conventionally defined as acute when it lasts less than 4 weeks [1]. It is more prevalent in younger children, particularly in pre-school age, compared to school-age children and adolescents [6]. Approximately two-thirds of children between 0 and 4 years old are brought to their pediatrician for an acute respiratory event [5]. An observational study by De Blasio et al. involving 433 children aged 1 to 14 years with acute cough found that 52% of the participants were under the age of 6 [70]. Furthermore, pre-school children have higher morbidity with worse impact on the QoL than children and adolescents who have shorter and milder episodes [71].

Coughing may be a presenting symptom of many diseases generally affecting the respiratory tract. Concerning acute cough, it is the presenting symptom of URTIs in approximately 90% of cases, mainly sustained by a viral pathogen, generally benign and self-limiting [6]. Young children frequently experience respiratory tract infections, largely due to increased exposure when they first enter communal settings [72]. Children may have up to 8–10 episodes of URTIs a year, and coughing may last for more than 2 weeks [73]. Before 2020, rhinovirus (RV) and common cold human coronaviruses were the most frequently identified microorganisms, followed by respiratory syncytial virus (RSV), human metapneumovirus, adenovirus, parainfluenza, and type A influenza viruses [74]. The SARS-CoV-2 pandemic interrupted that pattern, except for RV [75]. Other causes of acute cough may include croup [76], lower respiratory tract infections (LRTIs) such as pneumonia, bronchitis or bronchiolitis [77], rhinosinusitis [78], inhalation of a foreign body [5], wheezing or asthma [79]. Furthermore, acute coughing may be precipitated by exposure to allergens and irritants, such as exposure to tobacco smoke, second-hand exposure to e-cigarettes, pollutants, smells, aerosols, dust, or cold or dry air [6,73,80]. A recent meta-analysis by Galway et al. confirmed that exposure to tobacco smoke has a particularly harmful impact on children’s health and their respiratory systems [4].

The management of coughing in children should be based on a detailed history and an accurate physical examination. A helpful way to guide the diagnosis is to characterize the cough as wet or dry: most children present with acute wet cough that requires no investigations or treatment as it is caused by a self-limiting viral infection [5]. Moreover, some characteristics of the cough may be helpful in understanding its etiology: “barking cough” is typical of croup [76]; *Bordetella pertussis* infection causes a cough referred to as “paroxysmal” [81]; and a dry cough associated with specific respiratory signs such as wheezing and shortness of breath is mostly associated with asthma [79]. In the presence of any red flags, both systemic (failure to thrive, digital clubbing, neonatal onset, features suggestive of immunodeficiencies) and/or specific pulmonary signs (respiratory distress, dyspnea, cyanosis, hemoptysis, chest pain, specific lung sounds), the patient should be referred to a specialist and a chest X-ray and/or a lung function test should be performed [6]. In the absence of warning signs, no specific protocols are nowadays validated for acute cough in children and a “wait and see” approach should be preferred, as proposed by Marseglia et al. in a recent Italian review [6]. Firstly, it is essential to address parental concerns by reassuring them of the benign nature of the symptom in the absence of specific red flags and to identify and eliminate any environmental factors that may be exacerbating the condition [82]. In this regard, exercise is considered a recognized cough trigger that can occur with asthma or exercise-induced laryngeal obstruction [83].

Cough associated with URTIs frequently resolves within 10 days in approximately 50% of cases and in 90% of children within 25 days [84]. If URTI symptoms last more than 10 days, acute rhinosinusitis should be suspected [78]. Cough persisting for more than 4 weeks should be managed following specific chronic cough protocols [77]. The potential progression of LRTIs must be carefully monitored [77]. Routine antibiotic treatment is not recommended, as bronchiolitis or viral pneumonia could be part of the natural progression [77]. However, if risk factors for complications exist or if the patient is systemically unwell, antibiotics may be considered, and specialist consultation might be necessary [77]. For suspected inhaled foreign bodies, especially in cases of choking, acute dyspnea, or sudden onset of wheezing, referral to a specialist for rigid bronchoscopy and further diagnostic or therapeutic evaluations is essential [85]. While acute cough in pediatric patients is generally benign, it can cause significant anxiety for families, often leading them to seek specialist advice or resort to over-the-counter (OTC) medications and a “do-it-yourself” approach [86].

## 4. Chronic Cough

Chronic cough in children ≤ 14 years old is defined as a daily cough lasting more than 4 weeks [86,87]. A proportion of these patients may have persistent cough despite thorough investigation (idiopathic chronic cough) and treatment (chronic refractory cough) according to published guidelines [86].

The prevalence of chronic cough is difficult to define since there is no agreed definition in epidemiological studies [88]. According to reports in the literature, it seems that prevalence varies between 1 and 28% [89,90,91] with a peak of 9% in Eastern Europe [92], but to date, no studies have systematically compared the prevalence of chronic cough in children worldwide.

Concerning pathophysiology, different stimuli can increase the physiological cough reflex. As the most frequent cause of cough in children is represented by infections, a possible mechanism explaining the persistence of the cough reflex lies in the neuromodulation: microorganisms as viruses or inflammatory mediators can cause plasticity in the cough neural pathways, causing the cough to persist after the pathogen noxa is cleared [93]. still, cough itself could be a traumatic mechanical stress to the airway wall resulting in a self-perpetuating cough-reflex cycle by inducing neutrophilic airway inflammation and cough-reflex hypersensitivity [94]. Therefore, compared with adults, children present with different degrees of maturation of the airway morphology and of the neurological and immunological systems in the various pediatric age groups that influence the cough reflex, resulting in observed differences in etiology and management [95].

It is possible to recognize pulmonary and non-pulmonary causes of chronic cough in children; however, according to the most recent European guidelines [86], the three most frequent causes are asthma, PBB, and nonspecific cough that resolves spontaneously, usually due to post-infective conditions [86]. Marseglia et al. proposed an etiological classification of chronic cough by stratifying the pediatric population by age, resulting in a major incidence of asthma in infants and children > 2 years old; PBB in children 1–2 years old; unknown cause in children 2–5 years old, and psychogenic cough in adolescents [7]. Ioan et al. supported the idea that etiology in chronic cough in children is age-related [96]. For this reason, in children < 5 years old, the inhalation of a foreign body should always be considered, and due to the anatomical features of the child, airways anomalies such as tracheomalacia or tracheobronchomalacia must be considered [97,98]. In this context, PBB is a specific pediatric condition caused by typical respiratory bacteria (*Hemophilus influenzae*, *Streptococcus pneumoniae*, and *Moraxella catarrhalis*). If unrecognized, it may progress to a chronic suppurative lung disease until bronchiectasis. Diagnosis is based on four clinical criteria: chronic wet cough; no other symptoms associated with the cough; no evidence of an alternative diagnosis; and resolution of the cough after a two-week course of antibiotic treatment [99]. A dry cough associated with specific symptoms such as wheezing and a history of atopy is clearly indicative of asthma, whereas in some cases, asthma can be manifested only with isolated cough and is then called cough-variant asthma or cough-dominant asthma, a condition that has recently aroused the interest of many authors concerning its diagnostical and therapeutic aspects [100,101,102,103]. Among non-pulmonary causes of chronic cough in children, GERD seems to be not as frequent as in adults, so empiric treatments against this condition are not used in pediatric age when clinical features of GERD are absent [104,105]. Rather, psychogenic cough (or habit cough, tic cough or somatic cough disorder) is most common in school-age children and adolescents, with a male predominance [106]. Typical of this condition is the characteristic sound of the cough, usually barking or honking, and the absence of the cough once asleep [107,108,109].

Despite the cause, chronic cough is best seen as a symptom of an underlying disease in children and should be approached using pediatric-specific cough management protocols and valuable questionnaires, which may include the Cough-specific Quality of Life Questionnaire (CQLQ) and specific cough-related symptom questionnaires tailored for pediatric populations, to assess the frequency, duration, and impact of the cough on the child’s daily life, helping to characterize the nature of the cough and its potential triggers [86]. Studies and reviews over the past decade agree that using specific cough algorithms leads to earlier diagnosis and improved clinical outcomes, such as shorter duration of cough and increased QoL [7,86,110,111]. In this regard, Mukerji et al. supported the need for a multi-disciplinary approach and recourse to a specialist when a specific cause is identified [112]. The systematic evaluation of a child with chronic cough starts with a targeted detailed history, physical examination, chest radiograph, and, when applicable, spirometry, with the aim of categorizing the cough as specific (the etiology can be attributed to an underlying abnormality or disease) or nonspecific (no evidence of an underlying disease) [112]. Specific cough pointers include the nature of the cough (wet or dry), classically recognizable cough sounds (barky croup cough), auscultatory findings (such as wheeze), and associated conditions such as digital clubbing, failure to thrive (FTT), recurrent pneumonia, and abnormalities on chest radiograph or spirometry [85,86,112]. Conversely, a chronic cough is more likely to be nonspecific if it is dry and there are no abnormalities identified on initial evaluation. In this case, Marseglia et al. highlighted the importance of a “wait and see” approach, considering a post-infective self-limiting cough the primary cause [7]. However, if the cough persists, therapy should be started. In the case of dry cough, an inhaled corticosteroid (ICS), such as budesonide 400 ug/day for 2–4 week, is the first-choice treatment if a cough-variant asthma is suspected. In the case of dry cough without specific pointers, an antibiotic therapy, specifically amoxicillin–clavulanate for 2–4 weeks if PBB is suspected, should be started [4,7,113]. In the case of a resolution of the cough, the treatment may be stopped and the diagnosis is confirmed. In case of persistence, re-evaluating the patient is essential, searching for possible specific pointers that could simplify the management by addressing the patient to a specialist, whereas further investigations need to be undertaken, such as a complete blood cell count, erythrocyte sedimentation rate (ESR), C-reactive protein (CRP), and first-step immunity evaluation [7]. Otolaryngology visits and rhino-pharyngo-laryngeal fibroscopy could be useful, as well as a sweat test in the suspicion of cystic fibrosis [7]. Still, a blood culture, Mantoux test, serology or polymerase chain reaction (PCR) on nasopharyngeal secretions and sputum should be attempted in case of suspected infectious causes [7]. If the cough persists and all the tests are negative, it is mandatory to proceed with chest computed tomography (CT), fiber optic bronchoscopy, bronchoalveolar lavage (BAL), fractional exhaled nitric oxide (FeNO) testing, and pH-impedance and/or high-resolution esophageal manometry [7]. If an underlying condition is identified, the specific treatment must be started, whereas a psychological visit is mandatory in the suspicion of a habit cough [7].

The main differences between acute and chronic cough in pediatric age are summarized in Table 1.

## 5. Neuromodulators

Neuromodulation is the physiological process through which a neuron employs one or more endogenous chemicals, known as neuromodulators, to regulate a broad range of neurons or synapses in the nervous system [114]. In contrast to classical neurotransmission, where a neurotransmitter directly induces an excitatory or inhibitory response at the synapse, neuromodulation involves the release of substances that modify the intrinsic properties of neurons, the strength of synaptic transmission, or the overall excitability of neural circuits [114]. As a result, neuromodulation can shape various aspects of neuronal function, including synaptic plasticity, circuit dynamics, and behavior [115]. Neuromodulators encompass a wide variety of molecules, including monoamines (e.g., dopamine, serotonin, and norepinephrine), neuropeptides (e.g., substance P and oxytocin), and cytokines [116]. They can be released both synaptically and extrasynaptically, so they can operate locally or at distant targets, acting in a paracrine or endocrine way [116]. The effects of neuromodulators are typically mediated through G-protein-coupled receptors (GPCRs) rather than ion channels, leading to more sustained and widespread influences on neural activity [117]. G proteins are heterotrimeric complexes composed of α, β, and γ subunits. In its inactive state, the α subunit is bound to Guanosine Diphosphate (GDP) [117]. When an agonist binds to a GPCR, it triggers the exchange of GDP for GTP (Guanosine Triphosphate) on the α subunit [117]. This exchange induces a conformational change in the α subunit, causing its dissociation from the βγ dimer [117]. Both the activated α subunit and the βγ dimer then function as signal transducers, initiating various intracellular signaling pathways [117]. The complexity of these activation steps results in slower and more prolonged effects that can influence cellular responses across a broader area [115,117]. Specifically, the regulation of cough by neuromodulators is increasingly being explored in the last decades, both in pediatric and adult age [118]. In children, the cough reflex is more sensitive and easily triggered because of the ongoing neural development and the increased neuromodulator activity, such as substance P and neuropeptides, leading to a stronger response [118]. In contrast, adults have a fully developed nervous system with a more regulated cough reflex [118,119]. Additionally, the immaturity of the immune system in children may result in exaggerated inflammatory responses [118,119]. Overall, children have a more easily triggered cough reflex, while adults exhibit a more regulated response influenced by chronic conditions (e.g., Chronic Obstructive Pulmonary Disease (COPD), GERD, etc.) [118,119].

**Figure 2:**
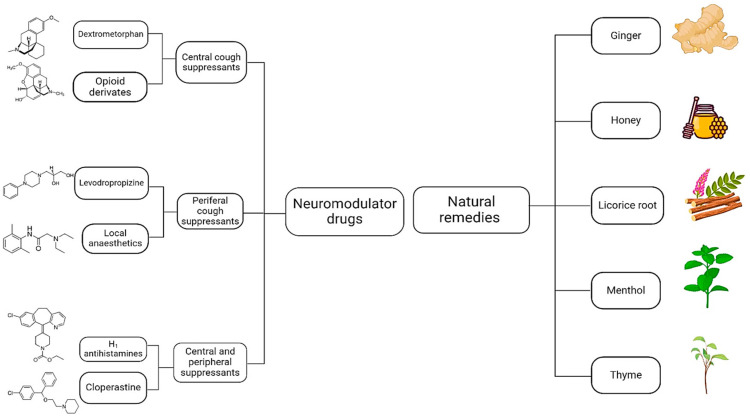
The major classes of neuromodulators for cough in pediatrics.

Neuromodulators are increasingly being explored as therapeutic options for managing acute and chronic cough in pediatric subjects, particularly in the case of absent or inadequate responses to conventional therapies; so, neuromodulators may influence both the sensitivity and the duration of the cough reflex [120]. As showed in Figure 2, they may be summarized into two major classes: neuromodulator drugs and natural remedies with neuromodulator effects.

### 5.1. Neuromodulator Drugs

Based on their mechanism of action, neuromodulator drugs may be classified into central, peripheral, and both central and peripheral agents [121].

#### 5.1.1. Central Cough Suppressants

**Dextromethorphan**

Dextromethorphan (DXM) hydrobromide acts by binding to several key sites in the brain, including N-methyl-D-aspartate (NMDA) glutamate receptors, sigma-1 (∂-1) receptors, as well as serotonergic and nicotinic receptors [122]. After being absorbed from the gastrointestinal tract, it undergoes metabolism in the liver, primarily by the cytochrome P450 (CYP) enzymes, particularly CYP2D6, converting it mostly into dextrorphan, its active metabolite with antitussive properties [123]. The effects of DXM can last for up to six hours [123]. The drug is primarily excreted through the kidneys [123]. The recommended dosage varies by age: 7.5 mg for children aged 2–5 years, 15 mg for those aged 6–11 years, and 30 mg for adolescents aged 12–18 years [124].

Currently, the American Academy of Pediatrics and the American College of Chest Physicians do not support the use of DXM for treating cough in children [125].

Regarding its efficacy, recently Meeves et al. conducted a multiple-dose, double-blind, placebo-controlled, randomized pilot trial to evaluate the antitussive efficacy of DXM hydrobromide in children aged 6–11 years with acute cough due to the common cold. The study included 128 children who first completed a run-in period with a cough monitor after taking a sweet syrup. They were then randomized to receive either DXM or a placebo for four days. Coughs were recorded over the first 24 h, and the children self-reported cough severity and frequency daily. The authors found that DXM significantly reduced total coughs over 24 h by 21.0% and daytime cough frequency by 25.5% compared to placebo. Children who received DXM also reported greater reductions in cough severity and frequency. However, no significant differences were observed between the DXM and placebo groups for night-time cough rates or the impact of cough on sleep. Both DXM and placebo were generally well-tolerated [126].

Regarding its safety, DXM can cause adverse reactions especially at higher doses or in sensitive individuals [127]. Common side effects include drowsiness, dizziness, confusion, nervousness, nausea, vomiting, stomach pain, and constipation [127]. Serious side effects, although rare, can include adverse drug reactions (ADRs), hallucinations and serotonin syndrome when taken with other drugs that increase serotonin levels (e.g., selective serotonin reuptake inhibitors (SSRI), tricyclic antidepressants, monoamine oxidase inhibitors, meperidine, lithium, clonazepam, methylenedioxy-methamphetamine (MDMA), and tryptophan dietary supplements) [127]. Toxicity has been observed at doses exceeding 10 mg/kg, with seizures reported at doses ranging from 20 to 30 mg/kg [128].

**Opioid derivates**

Primarily known for their analgesic properties, opioid derivates can act as potent neuromodulators in the management of cough [120].

Specifically, codeine, extracted from *Papaver bracteatum*, is a weak opioid that acts on the μ-opioid receptors in the medullary cough center [129]. Like other antitussive drugs, codeine is well absorbed after oral administration and metabolized in the liver by CYP450 and CYP2D6 [130]. Codeine and its metabolites are primarily excreted through the kidneys as free or glucuronide-conjugated forms [130]. It is available in the form of syrup, capsules, and tablets, in combination with other molecules or alone, in doses of 5–10 mg every 4–6 h as needed, and with a maximum daily dose of 60 mg [131].

In April 2015, the EMA issued a statement advising that codeine-containing cough and cold medicines should not be used in children under 12 years old [132]. The EMA also recommended caution in using these medications for children aged 12 to 18 years [132]. In 2017, the FDA released strong recommendations against the use of codeine in children, following reports of 24 deaths and 38 cases of respiratory depression linked to codeine-containing medications in pediatric patients [133]. In 2018, the FDA limited the use of these products to adults 18 years and older [134].

Regarding its efficacy, in a single RCT involving 57 pediatric patients with nocturnal cough (ages 18 months to 12 years), Taylor et al. compared the efficacy of codeine 10 mg/5 mL combined with guaifenesin 100 mg/5 mL, dextromethorphan, and placebo. Although codeine was associated with a reduction in mean cough and composite scores, these results did not achieve statistical significance and did not demonstrate greater effectiveness compared to either the other active treatments or the placebo [135].

Common side effects of codeine include drowsiness, constipation, and nausea [136]. More severe effects can include breathing difficulties and, in some cases, fatal respiratory depression, especially in children who are “ultra-rapid metabolizers” of the drug due to variations in the CYP2D6 enzyme, leading to potentially dangerous levels of morphine in the body [136].

#### 5.1.2. Peripheral Cough Suppressants

**Levodropropizine**

Levodropropizine is a piperazine compound derived from hydroxyzine, a first-generation antihistamine [137]. Rapidly absorbed by the digestive tract, with a plasma peak of 40–60 min after ingestion, levodropropizine undergoes hepatic metabolism, but it does not require activation via complex metabolic pathways such as CYP2D6 [138]. Approximately 80–90% of an administered dose is eliminated unchanged in the urine [138]. Levodropropizine acts primarily on the peripheral sensory nerves, specifically the C fibers, and inhibits the release of neuropeptides like substance P and bradykinin [138]. By blocking the release of these neuropeptides, levodropropizine reduces the activation of sensory neurons, leading to a decreased cough response [138]. This action is crucial in conditions where the cough is driven by peripheral irritation or inflammation rather than central nervous system (CNS) triggers [138]. Indeed, levodropropizine does not significantly affect the CNS [139]. Its selective action on peripheral receptors minimizes the risk of CNS-related side effects, such as drowsiness, sedation or respiratory depression [139]. This makes levodropropizine a safer option for treating cough in children, particularly for daytime use, as it does not impair alertness or cognitive function [139].

Levodropropizine is commonly indicated for short-term symptomatic treatment of acute cough in adults and children aged > 2 years old [140]. The dosage depends on patient’s age: 2.5 mg to 5 mg, administered three times daily, for children aged 2 to 5 years; 5 mg, administered three times daily, for children aged 6 to 12 years; and 10 mg, administered three times daily, for children over 12 years and adolescents [121].

Regarding its effectiveness, Zanasi et al. compared this drug to the efficacy of central cough suppressants (opioids and non-opioids), conducting a meta-analysis of seven clinical studies involving 1178 patients across pediatric and adult populations. They evaluated cough frequency, severity, and night awakenings as the outcome measures. The results showed that levodropropizine significantly outperformed control treatments in reducing cough intensity, frequency, and nocturnal awakenings. The findings confirm that levodropropizine is more effective than centrally acting antitussives like codeine, cloperastine, and dextromethorphan, offering a superior benefit–risk profile [141]. Recently, Marseglia et al. conducted a comprehensive literature review focusing on studies assessing the effectiveness of levodropropizine in managing acute post-viral cough in pediatric patients. The review synthesized data from RCTs and observational studies to provide a robust evidence base for the use of levodropropizine in this population. The analysis revealed that levodropropizine is effective in reducing cough frequency and severity in children and adolescents suffering from acute post-viral cough. Clinical studies demonstrated that levodropropizine significantly alleviates cough symptoms compared to placebo and other common cough treatments [121].

Concerning its safety, the medication is well-tolerated with a favorable safety profile. Rare and mild side effects, such as gastrointestinal discomfort or allergic reactions, may occur [120,121].

**Local anesthetics**

Local anesthetics (e.g., benzonatate, lidocaine) act by blocking voltage-gated sodium channels on sensory nerve fibers [142]. This inhibition prevents the initiation and propagation of action potentials in the nerves responsible for detecting and responding to irritants in the airways [142,143]. By blocking these channels, local anesthetics reduce the excitability of sensory neurons, leading to a decrease in the cough reflex. This mechanism is particularly useful in conditions where the cough is triggered by local irritation or inflammation [142,143]. These drugs are most used for inhibiting laryngeal and respiratory reflex in anesthetized children [144].

Benzonatate is administered orally and is systemically absorbed [30]. Although specific pediatric studies are limited, children, especially older than 10 years, metabolize benzonatate by esterases in the plasma to produce para-aminobenzoic acid (PABA) and other metabolites, excreted primarily through the kidneys [30]. Benzonatate typically acts within 15 to 20 min and provides relief for 3 to 8 h [30]. The usual dosage is 100 to 200 mg every 8 h, with a maximum daily dose not exceeding 600 mg [30]. Lidocaine can be administered through various methods, including intravenous, intramuscular, and topical routes [120]. Once administered, lidocaine is quickly metabolized in the liver through the CYP450 enzyme system, particularly CYP3A4 and CYP1A2, and excreted unchanged through the kidneys [120]. Dosage depends on the method of administration and the clinical indication [120].

Recently Hu et al. evaluated the effectiveness of different doses of intravenous lidocaine in reducing sufentanil-induced cough (SIC) in children. The study involved 188 pediatric patients (ages 3–12) scheduled for elective tonsillectomy with or without adenoidectomy. Patients were divided into four groups receiving different doses of lidocaine: Group A (0 mg/kg), Group B (1 mg/kg), Group C (1.5 mg/kg), and Group D (2 mg/kg). The primary outcome was the SIC grade during anesthesia induction. Secondary outcomes included the incidence of SIC and hemodynamic parameters (mean arterial pressure and heart rate). SIC grades were significantly lower in Groups C and D compared to Groups A and B. The incidence of SIC was highest in Group B and lowest in Group D. Severe cough incidence was notably reduced in Group D compared to the other groups. No significant differences were observed in hemodynamic parameters across the groups [145].

Regarding safety, serum concentrations of lidocaine > 5 mg/L may cause light-headedness, tremors, hallucinations, and cardiac arrest [146]. The side effect profile of benzonatate is considered relatively benign, but some patients may experience nausea, vomiting, or constipation, with mild drowsiness or dizziness occurring at higher doses [30,120].

#### 5.1.3. Drugs Acting Both Peripherally and Centrally on Cough Reflex

**H_1_ antihistamines**

Antihistamines are among the most prescribed drugs in pediatric care [147,148]. Specifically, H_1_ antihistamines inhibit the action of histamine on H_1_ receptors and can also inhibit α-adrenergic, muscarinic, serotonin receptors, and ion channels [120,148].

Anti-H_1_ antihistamines are functionally classified as first- and second-generation drugs [120,149]. First-generation H_1_ antihistamines are lipophilic, so they can easily cross the blood–brain barrier and enter the CNS, leading to sedative effects [120,147,148]. When administered orally, they typically reach peak concentration in children within 2 h and are then metabolized in the liver via the CYP450 enzyme system (primarily CYP2D6 and CYP3A4) and excreted in the urine [120,147,148]. In contrast, second-generation H_1_ antihistamines are lipophobic, which means they poorly penetrate the CNS, making them relatively non-sedating [120,147,148]. Furthermore, some second-generation H_1_ antihistamines also possess anti-inflammatory properties (e.g., reduction of cytokine release, inhibition of the synthesis of chemical mediators by mast cells and basophils) [120,147,148]. These drugs are also administered orally, with peak concentrations occurring between 30 and 60 min (e.g., cetirizine) or up to 3 h (e.g., astemizole) after administration in children [120]. Many second-generation H_1_ antihistamines undergo metabolism in the liver, primarily via the CYP450 enzyme system, while they are eliminated through the kidneys or in the feces, with some variation depending on the drug [120,147,148].

Regarding their efficacy, Ciprandi et al. evaluated the potential effectiveness of loratadine, a second-generation H_1_ antihistamine in treating allergic cough in 20 patients with allergic rhinoconjunctivitis and cough caused by *Parietaria judaica* during its pollen season. In this double-blind, placebo-controlled, parallel-group, randomized study, allergic patients were given either loratadine 10 mg/day or a placebo in oral tablet form for 4 weeks. Patients recorded the occurrence and severity of conjunctival and nasal symptoms, as well as the frequency and intensity of cough attacks, daily. They also measured peak expiratory flow (PEF) twice daily, in the morning and evening. Physicians assessed conjunctival and nasal signs, and performed spirometry at the start of the study, and again at 2 and 4 weeks after treatment. A methacholine challenge was conducted at the beginning of the study to assess PD20. Pollen counts were monitored throughout the study. Loratadine treatment led to a significant reduction in ocular and nasal symptoms, as well as a decrease in cough frequency and intensity. In the placebo group, peak expiratory flow rate, forced vital capacity, and forced expiratory volume in one second showed a significant decline, while these measures remained stable and within normal ranges in loratadine-treated patients. These findings suggest that loratadine may be effective in treating allergic cough and rhinoconjunctivitis [149]. A systematic review by Smith et al., which included three RCTs examining the effectiveness of antihistamines in treating acute cough, found that antihistamines were no more effective than placebo in reducing cough frequency or improving sleep-related outcomes [150]. More recently, Wei et al. evaluated the clinical efficacy of combining montelukast sodium with either budesonide or loratadine in treating children with cough variant asthma. A total of 72 pediatric patients were divided into two groups: Group A (*n* = 35) received montelukast sodium combined with budesonide, while Group B (*n* = 37) received montelukast sodium combined with loratadine. The study assessed the clinical efficacy of both treatment combinations by measuring lung function indices: forced expiratory volume in the first second (FEV1), the ratio of FEV1 to forced vital capacity (FEV1/FVC), and PEF. Additionally, inflammation biomarkers (TNF-α and IL-4), eosinophil levels, and IgE levels were monitored at three intervals: before treatment, at 4 weeks, and at 12 weeks post-treatment. The study also recorded adverse reactions, symptom recurrence, and treatment compliance. The results showed significant improvements in lung function, inflammation markers, eosinophil levels, and IgE levels in both groups after treatment. However, Group A had lower treatment compliance compared to Group B. In conclusion, both treatment combinations are clinically effective in managing cough variant asthma, improving lung function and reducing inflammation with minimal adverse reactions and low recurrence rates. Both methods are recommended for clinical use [151].

Concerning safety, first-generation H_1_ antihistamines often cause CNS effects, such as drowsiness, fatigue, increased appetite, and impaired cognitive function. Additionally, they can exhibit antimuscarinic, antiadrenergic, and anti-serotonergic properties, potentially leading to side effects like dry mouth, tachycardia, vision disorders, and confusion [147,148]. Second-generation antihistamines were associated with serious cardiac side effects, including significant QT interval prolongation and severe arrhythmias, such as life-threatening torsade de pointes [152].

**Cloperastine**

Cloperastine is an antitussive agent that acts directly on the cough center in the brainstem, reducing the cough reflex and thereby decreasing cough frequency, without causing respiratory depression or impacting the cardiovascular system [153]. Due to its structural similarity to H_1_ receptor antagonists, cloperastine also presents antihistaminic properties and exhibits papaverine-like activities [154]. It is taken orally and begins to act typically within 20–30 min after administration, with effects lasting for 3–4 h [120,154]. Cloperastine undergoes hepatic metabolism via the CYP450 enzymes and is primarily excreted unchanged in the urine [154].

It is generally considered safe for use in children, but its use is often limited to those aged 2 years and older [120]. The dosing should be carefully adjusted according to the child’s age and weight and varies depending on the formulation (e.g., syrup, tablets) and the age of the child [120].

Regarding its efficacy, Scotti et al. conducted a double-blind controlled trial in children suffering from various cough-inducing conditions. The findings suggest that cloperastine is effective in reducing cough frequency and severity without significant side effects [155]. Svitaylo et al. conducted a randomized, double-blind, placebo-controlled study to evaluate the efficacy and tolerability of cloperastine in treating nonproductive cough in 200 pediatric subjects. The study specifically focused on the drug’s impact on night-time cough and the quality of sleep for both patients and their parents. The results indicated that cloperastine significantly reduced night-time cough frequency, leading to improved sleep quality for both the patients and their parents [156].

Common side effects of cloperastine include drowsiness, dry mouth, and dizziness due to its antihistamine properties [153]. Less commonly, gastrointestinal disturbances (e.g., mild and transient nausea) may occur [153].

### 5.2. Natural Remedies with Neuromodulator Effects

Several natural products, grounded in historical and cultural practices, are commonly employed across different societies to alleviate cough symptoms [157]. All these compounds share the same pharmacokinetics, with absorption in the gastrointestinal tract, metabolism in the liver, and excretion through urine [157].

**Ginger**

Ginger (*Zingiber officinale*) holds promise as a natural remedy for cough in children due to its anti-inflammatory, antioxidant, and antimicrobial properties [157]. It contains bioactive compounds such as gingerols and shogaols, which have anti-inflammatory and neuromodulatory properties and may help reduce cough by inhibiting the production and release of pro-inflammatory mediators and modulating neural pathways [157,158].

To the best of our knowledge, no studies evaluating the efficacy and safety of ginger were so far conducted in pediatric age and its use is currently not recommended for the treatment of cough in children [158].

**Honey**

Honey is a nutritious, natural, and healthy food produced by honeybees (*Apis mellifera*) [159]. Generally, it has a content of 80–85% carbohydrates, 15–17% water, 0.3% proteins, 0.2% ashes, and minor quantities of amino acids, phenols, pigments, and vitamins [160].

Honey has bactericidal, anti-inflammatory, antioxidant, metabolic, and antitussive properties [161]. Its viscosity increases saliva production and swallowing, which sends an irritative stimulus to the cortical neural network [120]. It interferes with the cough reflex, reducing the sensitivity of cough receptors and the frequency and severity of cough, particularly at night [120].

Honey is commonly used to treat acute cough in children over one year old [162]. It should not be given to infants under one year due to the risk of infection caused by *Clostridium botulinum* [162].

Anibasa et al. investigated the efficacy of honey in treating cough caused by URTIs in children, compared to diphenhydramine, assessing the impact of honey on cough frequency, severity, and the associated stress on caregivers. A single-blind RCT was conducted with 84 children who had URTI-related cough. The children were randomly assigned to receive either honey (intervention group, *n* = 42) or diphenhydramine (control group, *n* = 42) over three consecutive nights. Data on cough frequency, severity, and caregiver stress were collected using the Pediatric Cough Questionnaire and Kingston Caregiver Stress Scale. The analysis showed that honey significantly reduced cough frequency and severity compared to diphenhydramine. Additionally, caregivers of children in the honey group reported a greater reduction in stress and improved sleep patterns for both themselves and the children [163].

Nishimura et al. assessed the effectiveness of honey in treating nocturnal coughs and sleep disorders in young children with URTIs. They conducted a multicenter, randomized, double-blind, placebo-controlled trial in Japan, including 161 children aged 1–5 years who had URTIs and coughs lasting up to 7 days. Participants were recruited from 13 pediatric community clinics and randomly assigned to receive either acacia honey or a honey-flavored syrup placebo. The treatments were administered before bedtime on two consecutive nights, and nocturnal cough and sleep difficulties were assessed using a 7-point Likert scale. Data collection occurred from November 2021 to February 2022, with 78 children in the honey group and 83 in the placebo group. Both groups experienced improvements in cough and sleep difficulties over the two nights, but there were no significant differences between the honey and placebo groups [164].

More recently, Kuitunen et al. conducted a systematic review to evaluate the efficacy and safety of honey as a treatment for acute cough in children. The authors analyzed multiple studies to determine whether honey is a viable alternative to conventional cough medications. Honey was found to be effective in reducing cough frequency and severity, improving sleep quality for both children and their parents. It was especially beneficial compared to placebo or no treatment. Furthermore, honey is generally safe for children over one year of age, with minimal side effects reported. The authors suggest that honey performs similarly or better than OTC cough suppressants and is a cost-effective option for managing acute cough in children. The study concludes that honey is an effective and safe option for treating acute cough in children, supporting its use as a natural remedy in pediatric care [165].

**Licorice root**

Licorice (*Glycyrrhiza glabra*) has a long history as a remedy for coughs and respiratory disorders [157,166].

Kuang et al. assessed the effectiveness of 14 key compounds and crude extracts from licorice using two experimental models in mice: the ammonia-induced cough model and the phenol red secretion model. The authors found that liquiritin apioside, liquiritin, and liquiritigenin, when administered at 50 mg/kg orally, significantly reduced cough frequency by 30–78%. The antitussive effects of these compounds were partially blocked by methysergide or glibenclamide, but not by naloxone, indicating that their action involves both peripheral and central pathways. These compounds also exhibited potent expectorant effects after a 3-day treatment. Water and ethanol extracts of licorice, rich in liquiritin apioside and liquiritin, reduced cough frequency by 25–59% at 200 mg/kg and enhanced phenol red secretion. In contrast, ethyl acetate extracts were less effective [166].

Recently, Rabbani et al. conducted a randomized placebo-controlled trial to assess the efficacy of an oral combined tablet containing *Glycyrrhiza glabra*, *Viola odorata*, and *Operculina turpethum* as an adjunctive therapy for managing mild-to-moderate childhood asthma. A total of 60 children and adolescents with chronic mild-to-moderate asthma were assigned to either the intervention group or the placebo group, in addition to their standard asthma treatment. Outcomes were measured through clinical assessments of cough frequency and severity, shortness of breath, spirometry indices, disease control, and treatment adherence using validated questionnaires. Patients in the first group showed significant improvements in respiratory test indices and Asthma Control Questionnaire scores and a reduction in the severity of activity restriction compared to the placebo group. Notably, there were significant differences in the frequency and severity of coughs and activity restriction. The Asthma Control Questionnaire scores also improved significantly for the Anti-Asthma^®^ group compared to the placebo [167].

Currently, the EMA does not recommend its use in subjects under 18 years old [168].

**Menthol**

Menthol is an acyclic monoterpene derived from the peppermint plant (*Mentha piperita*) [169]. It exhibits a range of therapeutic properties, including analgesic, anti-inflammatory, antitussive, antiviral, and antifungal effects [120]. These benefits are primarily attributed to its interaction with nociceptors, such as TRPM8, which are predominantly found on afferent sensory neurons [120].

Clinical evidence of menthol’s efficacy is minimal and based on small cohorts of patients. Kania et al. conducted a single-blind, pseudo-randomized crossover trial with 42 healthy children, aged 10–11, to evaluate the effects of menthol on nasal airflow, perceived nasal openness, and cough response. Participants inhaled either menthol or a placebo (eucalyptus oil), and measurements were taken before and after intervention on two consecutive days. The study found that menthol did not affect nasal airflow as measured by spirometry. However, menthol significantly increased the children’s perception of nasal openness. Although menthol reduced cough count slightly, this change was not significantly different from the placebo [170]. 

Mild, temporary irritant effects (e.g., burning sensation on the skin, eyes, or nose) may follow menthol administration [120]. More severe adverse events, like seizures related to dermal exposure, are typically limited to young infants [120].

The use in children under 2 years of age is contraindicated by the EMA, while it is not recommended in children between 2 years and 11 years of age [171].

**Thyme**

Thyme (*Thymus vulgaris*) contains thymol and carvacrol, which have antimicrobial, anti-inflammatory, and antitussive effects. These compounds may act on neuromodulatory pathways to reduce cough reflex sensitivity [172].

Recently, Eskandarpour et al. conducted a randomized, triple-blind clinical trial to evaluate the effect of *Thymus vulgaris* on cough in children aged 5 to 12 years experiencing mild to moderate asthma exacerbations. A total of 60 children were divided into two groups: the intervention group (*n* = 30) received *Thymus vulgaris* (20 mg/kg every 8 h) in syrup form along with standard medical treatment for one week; the control group (*n* = 30) received standard medical treatment with placebo syrup. Clinical symptoms, laboratory measures, and spirometry data were assessed before and after the intervention. The intervention led to a significant reduction in activity-induced cough in the first group compared to the control group. However, there was no significant difference in the reduction of wheezing and breathlessness between the groups. Spirometry results showed a significant improvement in FEV1 in the intervention group, but there were no significant differences in FEV1/FVC, PEF, or forced expiratory flow at 25–75% of vital capacity (FEF25–75%) [172].

Currently, its use is not recommended by the EMA in subjects under 18 years old [173].

## 6. Conclusions

The exploration of neuromodulators in the field of acute and chronic cough in children provides significant insights into the unique underlying mechanisms that differentiate pediatric cough from adult presentations. Neuromodulators can play a critical role in the increased sensitivity and persistence of cough frequently observed in children. These findings highlight the need to consider age-related differences in neuromodulation pathways when diagnosing and treating pediatric cough, as children’s neural pathways can respond differently to stimuli compared to adults.

Understanding these mechanisms not only enhances our knowledge of cough pathophysiology, but also opens new avenues for targeted therapies that could improve the outcomes of children suffering from both acute and chronic cough.

Further research in this area is critical. A deeper exploration of how neuromodulators interact with the pediatric cough reflex will provide a clearer understanding of the long-term effects and safety of these treatments, significantly improving the QoL for children, especially those suffering from persistent or recurring cough, and their caregivers. The potential for such advances makes this a vital area of ongoing investigation.

## Figures and Tables

**Table 1:** A summary of the main differences between acute and chronic cough in pediatric age.

	Duration	Etiology	Evaluation
**Acute cough**	<4 weeks	URTIs Croup LRTIs Ambiental agents’ exposition	As acute cough is usually self-limited, a “wait and see” approach is preferred
**Chronic cough**	>4 weeks	PBB (children aged 1–2) Asthma (children aged >2) GERD, asthma, post-infective, psychogenic cough (adolescents)	As chronic cough is often a symptom of an underlying disease, pediatric-specific cough management protocols should be used

(GERD: gastroesophageal reflux disease; LRTIs: lower respiratory tract infections; PBB: protracted bacterial bronchitis; URTIs: upper respiratory tract infections).

## Data Availability

No new data were created or analyzed in this study.
